# Nutrigenomics: Definitions and Advances of This New Science

**DOI:** 10.1155/2014/202759

**Published:** 2014-03-25

**Authors:** N. M. R. Sales, P. B. Pelegrini, M. C. Goersch

**Affiliations:** ^1^Universidade Anhembi Morumbi/Gerência de Nutrição Enteral e Parenteral, Rua Pedro Ivo 22, 01323-070 São Paulo, SP, Brazil; ^2^BioLife Brasil Ltda, Distrito Agroindustrial de Formosa, Quadra 01, Lote 15, 73801-970 Formosa, GO, Brazil; ^3^Gerência de Nutrição Enteral e Parenteral-GANEP, Rua Pedro Ivo 22, 01323-070 São Paulo, SP, Brazil

## Abstract

The search for knowledge regarding healthy/adequate food has increased in the last decades among the world population, researchers, nutritionists, and health professionals. Since ancient times, humans have known that environment and food can interfere with an individual's health condition, and have used food and plants as medicines. With the advance of science, especially after the conclusion of the Human Genome Project (HGP), scientists started questioning if the interaction between genes and food bioactive compounds could positively or negatively influence an individual's health. In order to assess this interaction between genes and nutrients, the term “Nutrigenomics” was created. Hence, Nutrigenomics corresponds to the use of biochemistry, physiology, nutrition, genomics, proteomics, metabolomics, transcriptomics, and epigenomics to seek and explain the existing reciprocal interactions between genes and nutrients at a molecular level. The discovery of these interactions (gene-nutrient) will aid the prescription of customized diets according to each individual's genotype. Thus, it will be possible to mitigate the symptoms of existing diseases or to prevent future illnesses, especially in the area of Nontransmissible Chronic Diseases (NTCDs), which are currently considered an important world public health problem.

## 1. Introduction

**Figure 1:**
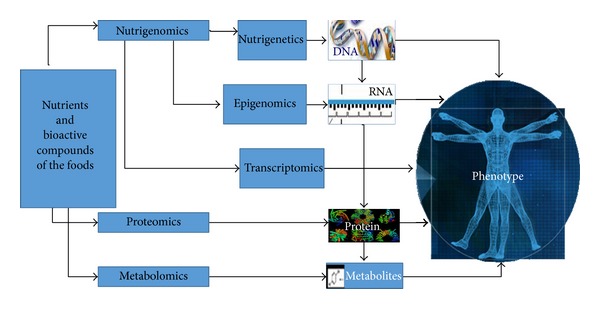
“Omics” sciences used in understanding the relationship between nutrition versus health versus disease (source: [4], with modifications; [9] with modifications).

Food intake and the environment are the two main factors that affect the health or illness of an individual [1]. Studies in nutritional area have increased the understanding of how to maintain healthy a group of individuals that live in different dietary conditions [2–4]. However, after the conclusion of the Human Genome Project (HGP), new insights about the influence of nutrients into people's diet were postulated, which included (i) will gene expression in response to metabolic process, at cellular level, influence the health of an individual? (ii) Are gene expression and metabolic response the result of the interaction between genotype and environment/nutrient? (iii) Understanding how this interaction process occurs between gene and nutrient could lead to the prescription of specific diets for each individual. Hence, in order to answer those questions, Nutrigenomics was introduced [2, 5–7]. The studies on Nutrigenomics are focused on the effects of the nutrients over the genome, proteome, and metabolome, as illustrated on Figure 1 [2, 7–10].

Therefore, Nutrigenomics is the area of nutrition that uses molecular tools to search, access, and understand the several responses obtained through a certain diet applied between individuals or population groups [2, 5, 11, 12]. It seeks to elucidate how the components of a particular diet (bioactive compound) may affect the expression of genes, which may have increased its potential or which can be suppressed [2, 5, 12]. This response will depend on how genes will show a changed activity or alter gene expression (Figure 1) [2]. Some examples of this gene-nutrient interaction are their capacity on binding to transcription factors. This binding enhances or interferes with the ability of transcription factors on interacting with elements that will lead to the binding control of RNA polymerase. Earlier studies performed with vitamins A, D and fatty acids have shown that they can trigger direct actions in activating nuclear receptors and induce gene transcription [5, 13, 14]. Compounds such as resveratrol present in wine and soy genistein may indirectly influence the molecular signaling pathways, such as the factor kappa B [8, 13, 14]. The involvement of these factors in the activation and regulation of key molecules is associated with diseases ranging from inflammation to cancer [13, 14].

With information obtained from the HGP, it was found that humans have 99.9% identity between their genomes. A distinct difference between their weight, height, eye color/hair, and other features is only 0.1% of the gene sequence and this difference, among other factors, also determines the nutritional requirements and the risk of developing some of the NTCDs [2, 3, 16]. Single Nucleotide Polymorphisms (SNPs) are the main reason for this genetic variation, and it can often change the encoded protein [5, 17]. Studies have shown that certain genes and their variants can be regulated or are influenced by nutrients/food compounds from the diet and that these molecular variations may have beneficial actions to the health of an individual [2, 3, 17, 18].

### 1.1. From Nutrition to Nutrigenomics

Research on nutritional area can be seen since 400 BC, when Hippocrates speculated the hypothesis that the warm body temperature was innate. Around 1700 AD, the so-called “analytical Chemistry Era” began [12]. During this period, Lavoisier discovered how food was metabolized by the body, generating water, carbon dioxide, and energy [19]. In the 19th century, Liebig identified carbohydrates, proteins, lipids, and other macronutrients that released heat [12, 19]. During the next Era, called “Chemical and Analytical Era of Nutrition”, which occurred between the 18th and 20th centuries, Antonie Lavoisier made important discoveries on food metabolism and their relation with energy production, including its relevance on breathing and oxidation [12, 19]. Later, during the “Biological Era”, as the 19th century is known, studies on metabolism and chemistry were done, helping the science of nutrition on defining their role in the development and prevention of chronic diseases, such as cancer, cardiovascular, neurodegenerative, and bone metabolism disorders [12, 19]. Nowadays, the “Pos-Genomic Era” is being experienced. This era is characterized by the integration of three fields: biological, social, and environmental, where scientific discoveries on nutritional pathophysiology and metabolism are included [12, 19].

### 1.2. Nutrigenomics and Other Omics Sciences

After the HGP, discussions and actions began on a new biological era, the “Post-Genomic Era”, where the evolution of bioinformatics provided advances in “omics” science research. These sciences use biotechnology to isolate and characterize a greater number of biomolecules from the same group, such as DNA, RNA, proteins, or metabolites. Hence, after Genomics, other Omic Sciences appeared as revolutionary tools, such as Proteomics, Metabolomics and Transcriptomics [8, 20].

Therefore, the first definition of Nutrigenomics referred only to studies on the effects of nutrients/bioactive food on gene expression of an individual. Nowadays, this definition expanded and, recently, Nutrigenomics also involves the studies on nutritional factors that act protecting the genome. Thus, this new science seeks to understand the influence of dietary components on the Genome, Transcriptome, Proteome, and Metabolome [1–3]. Nutrigenomics, in a single experiment, can generate multiple responses, so it is necessary to be innovative in the approaches of this area [2]. Moreover, Nutrigenomics is able to extract useful biological information from the data collected. The final answer can only be achieved after a series of investigations or surveys among different groups and teams. Therefore, there is a growing partnership between countries/teams/research groups, involving Nutrition field, Biology, Medicine, Genomics, and Bioinformation [1, 4, 5].

### 1.3. Nutritional Epigenomics

Epigenomics can be defined as the study of the complete set of epigenetic modifications in a cell or in a tissue at a given time [6, 21]. The epigenome consists of chemical compounds that modify or mark the genome in such way that it can indicate what a cell can do and where and when to do it. These marks are called epigenetic marks [6]. These epigenetic marks are passed from one cell to another when they divide themselves and thus will be passed from generation to generation. These signatures are influenced by genotype in the surrounding media (environment, diet, and drugs, e.g.) and will determine the phenotype (Figure 1) [6–9].

### 1.4. Epigenetics

The epigenetic studies the modification of DNA and proteins, linking the DNA and histones, which may cause changes in chromatin structure without changing the sequence of the nucleotides [7, 10]. Epigenetics is the transmitted information based on gene expression as its changes begin slowly but are progressive and potentially reversible [7, 11]. An example is the folate metabolism, where folic acid, which is a nutrient related to the genetic integrity, ensures a balanced amount of deoxyribonucleotides for DNA replication [2, 11]. It acts as a cofactor for enzymes associated with the biosynthesis of nucleotides and thymidylate (protein found in the RNA molecule), as universal donor of methyl and DNA methylation reactions [7, 11].

Modulations in gene expression may be caused by epigenetic mechanisms through changes in chromosome structure [4, 12]. Examples are DNA methylation and histone acetylation. Earlier studies have shown that DNA methylation is directly related to the remodeling of chromatin and this, in turn, is induced by the nutrient enzyme DNA methyltransferase (DNMT), which catalyzes the transfer of a methyl group from S-adenosylmethionine to specific sites on the DNA [7, 12]. The S-adenosylmethionine metabolizes compounds from food as folic acid, vitamins B6, B12, B2, choline, and methionine [2, 12]. A deficiency of these can lead to changes in carbon metabolism and thus impair DNA methylation, increasing the risk of development of NTCD. The DNA hypermethylation suppresses the gene responsible for transcription, once hypomethylation is associated with malignancies, such as prostate cancer and hepatocellular [4, 12].

### 1.5. Nutritional Transcriptomics

Transcriptomics studies the complete set of activated RNA transcripts [11]. The mRNAs are produced by a given moment and in a given tissue of a selected organism; therefore, gene expression varies according to the different circumstances and periods of time [8, 11]. Transcription factors, when activated, migrate to the nucleus and bind to a specific sequence of DNA in the promoter region of genes and, there, act by inhibiting or facilitating transcription [4, 8]. These transcription factors can be stimulated by (i) physiological signals, such as those triggered by nutrients/bioactive food compounds or the metabolites resulting from them; (ii) hormones, pharmacological treatments, and diseases, among others [8, 13]. They act as sensors regulating/modulating transcription of the cells as needed [13]. In nutrition research, transcriptomics can assist in providing information about the mechanisms or underlying effects of a particular nutrient or diet. It can also help identifying genes, proteins, or metabolites that change in the state of prediseases, as well as assisting on recognizing and characterizing the pathways regulated by nutrients or bioactive compounds in foods [8, 9, 11, 13].

### 1.6. Proteomics

Proteomics is the science that studies the complete set of proteins involved in the biological processes of a certain species [11, 21]. These proteins act in the cell, tissue, or organ in its normal state, but in different physiological or pathological situations, they may change their expression level, or even their activity, likewise in transcriptomics [8, 11, 21].

Proteins are an important class of molecules that are found in all living cells. They play a variety of roles in the cell such as structural, mechanical, biochemical, cell signaling, transport, and storage [2, 6]. They are also an essential part of the human diet [2, 6]. The number of proteins produced by an organism is much larger than the number of genes that it possesses. This happens due to the numerous posttranscriptional/translational modifications. Proteomics uses a set of technologies designed to study the expression of proteins. For this, it utilizes strategies, such as chromatographic techniques associated with electrophoresis, prefractionation of samples by extraction sequences, and organellar proteome analysis, among others [3, 4, 8, 14]. Hence, Proteomics is a primordial resource for Nutrigenomics, once that it is the gap between genome sequences and cell behavior, becoming the biological tool used to understand the process of genetic function determination, and of how genome is activated in response to certain diet [3, 4, 8, 14]. An example is the activity of butyrate, which can alter the expression of several proteins from the ubiquitin-proteasome system. This alteration suggests that proteolysis can be the mechanism by which butyrate can regulate key-proteins on the control of cell cycle, apoptosis, and cell differentiation [4].

### 1.7. Metabolomics

Metabolome consists of a set of small primary/secondary metabolites and body fluids of an organism or species. Metabolomics is the area of functional genomics that studies the changes in metabolites, whose goal is to isolate and characterize them [1, 3, 22]. Research advances in this area may facilitate the understanding of how the genotype is related to the phenotype of an individual. Nutritionally, metabolomics has many applications, once that it allows knowing the arrangements and metabolic disorders caused due to a person's diet and how these changes may affect the one's health or disease [1, 3, 8, 16, 22]. Hence, metabolomics also studies the metabolism under environmental and genetic perturbations [11], which can be analyzed and interpreted with the help of bioinformatics and statistical tools [11, 17].

The metabolites are dissolved in the cell cytosol and are small organic molecules that interact directly with the proteins and other macromolecules. They are divided into primary and secondary metabolites [1, 11, 22]. Primery metabolites are directly involved with the routes of synthesis and degradation of macromolecules, while secondary metabolites are more common in plants and fungi, and act as structural components and their defense [3, 22]. The metabolites in living beings can act as substrates, such as inhibitors or activators of an enzyme, molecular precursors, wastes of synthesis, or degradation of macromolecules, among others [3].

In the area of nutrition, metabolomics allows the understanding of metabolic arrangements and instabilities that are the cause or suffer interference from the diet [1, 11]. This contributes to the knowledge of how the excess or lack of some nutrients or compounds (secondary metabolites) present in food can affect the health/illness of an individual. These compounds (nutrients or not) interact in several ways within the body, changing the metabolome pathways. As an example, perilla alcohol (monoterpene extracted from strawberry) can act as an anticancer molecule under certain organic stimulations [1, 3].

### 1.8. Nutrigenomics and Nontransmissible Chromic Diseases (NTCDs)

Nutrition is the process that offers different substances to an organism that can work as energy supplier (carbohydrate and fat), as cell structure sources (proteins), and on metabolism control (vitamins and minerals), thus maintaining its homeostasis [16]. The nutritional state of an individual is the result of the interaction between various factors, such as genetic background, physical body, and emotional and social state [4]. Diet is a key role factor, once those nutrients and other bioactive compounds present in food can either be beneficial or initiate several diseases [4, 8, 16]. Among the illnesses related to food consumption, there are the celiac disease, phenylketonuria, and NTCDs, such as cancer, diabetes, and dislipidemies [8]. In this way, the health state of a person will depend on the interaction between their genes and their food diet [11]. Therefore, Nutrigenomics, along with other omic sciences, aim to clarify the interaction between genes and bioactive compounds from food sources [4, 8, 11, 12, 16, 17].

### 1.9. Obesity

Researches have indicated that not only environmental factors, but also genetics aspects are related to health problems, including DCNTs and Metabolic Syndromes (MS) [8, 23]. Recent studies showed that 80% of the difference observed in the body mass index (BMI) of twins are related to genetic factors [23]. As obesity causes a chronic process of inflammation, the use of Nutrigenomics to modulate this manner is highly promising [8]. Other reports demonstrated that some food contain anti-inflammatory bioactives, such as the caffeic acid (found in Yerba mate), tyrosol (found in olive oil), quercetin (present in fruits and greeneries), and lycopene (present in tomatoes, guavas, and watermelon). These molecules act inhibiting the expression of COX2 and iNOS genes through the reducing the translocation of the Kappa-B nuclear factor from the cytoplasm to the nucleus [8, 16].

There are several other ways where bioactive compounds from food can interfere on genes. One of the primary mechanisms for gene expression modulation is during transcription [2, 4], where the synthesis of inflammatory mediators occurs and has a fundamental role on numerous chronic illnesses, including obesity [2, 4, 24]. Hence, interleukin-1 is one of these mediators, which, after activation, stimulates the production of many other molecules during the inflammation cascade. The bioactive compound *α*-tocoferol, found in green tea, acts by decreasing the level of this chronic inflammatory process that occurs in obese individuals. Therefore, studies indicate that this component can assist on the treatment of obesity [2, 24].

### 1.10. Cancer

The need for certain micronutrientes by the organism depends on the person's age, genetic background, and physical state [4, 25]. Earlier studies showed that the deficiency of micronutrients, such as folic acid, vitamins B12, B6, C, and E, selenium, niacin, and zinc can cause changes into the DNA similar to what is seen after radiation exposure [2, 4, 25]. These alterations can lead to the rupture of the DNA double strand, oxidative lesions, or both. Furthermore, they demonstrated to be narrowly related to the development of cancer [25].

Moreover, molecules present in contaminated food can produce toxic metabolites that may interact with DNA, modifying its structure and inducing mutations. It is the case of aflatoxin B1, which forms an adding compound able to bind to the N-7 position of guanine residue, generating a new product. This new molecule cleaves, then, the interaction between one sugar and one nitrogenous base of a nucleotide, leading to the formation of an apurinic site. The mutation can, thus, cause severe damages on the liver, including necrosis, cirrhosis, and carcinoma [7, 14].

During the metabolism of folate, the folic acid found in food sources, is absorbed by the intestine and, through many chemical processes of catabolism and synthesis, it is transformed into 5-methyltetrahydrofolate. This chemical component is necessary for the synthesis of methionine, which in turn is used during the process of DNA mutilation. Thus, a diet poor in folic acid can alter this process and interfere on DNA replication, leading also to an increased risk of cancer development [2–4, 25].

Nevertheless, various minerals work as protectors against cancer development [25]. Among them, there are (i) selenium, which stimulates the production of glutathione peroxidase enzyme that acts on the reduction of hydrogen peroxide and maintain the integrity of cell membranes; (ii) prostacyclins, which decrease the oxidative damage of important molecules, such as DNA, lipids, and lipoproteins; (iii) zinc, which act on processes for the maintenance of genomic stability, genetic expression, and apoptosis modulation [3, 25].

### 1.11. Type II Diabetes

Diabetes counts for more than 90% of all diseases of the world [2]. Type II Diabetes is a multifactorial pathogenesis that involves the interaction between genetics and environmental factors [23]. Genomics studies showed that there are 65 SNPs associated with the risk of developing type II Diabetes [26].

With the advances of genome sequencing and the decoding of the human genome, tests for the detection of SNPs related to Type II Diabetes became available to the public. In these exams, the patient is able to know it there is a genetic predisposition to develop the disease [26]. However, caution must be taken on applying this test for clinical practice, once that studies have shown that patients that had negative results on this exam for the presence of Type II Diabetes SNPs felt so secure about it that they stopped taking care of their food diets. Consequently, some later developed Diabetes due to food income and insulin tolerance [26]. Nevertheless, patients who received a positive result for the presence of type II Diabetes change their life style, especially in their food intake, which later decreased the development of the disease by this group [26].

## 2. Conclusion and Perspectives

Nutrigenomics shows a new way of working with nutrition and now, the knowledge of how food interferes with the genetic code and how the organism responds to these interferences and with the phenotype can be clarified.

Furthermore, the development inside Nutrition Sciences, together with communication and marketing fields, provided the emergence of a personalized nutritional counseling based in Nutrigenomics. After analysis, it was observed that nine different models can be built (Table 1).

Moreover, other sophisticated programs are available, where it is even possible to obtain a nutritional counseling based on the client's DNA. In this way, Gollust et al. [27] published a report where he studied the intention of patients on using online hypothetic programs for personalized nutritional counseling. During the research, the patients described that the presence of a physician during the nutritional evaluation and the compilation of data into the program was essential to bring a trusting diet plan and nutritional counseling through the online system [27].

Furthermore, in order to evaluate the impact of personal genomics to the population, a study placed in The United States of America intended to analyze the motivation, knowledge, risks, and benefits of sharing personal data to build a personalized field of every individual's health condition [27]. Hence, it was observed that 92% of the interviewed ones were willing to share their personal information to professional of health area, while 32% considered themselves unaware of what a genomic evaluation could do to their personal data [27].

Henceforward, the recent advances on Nutrition Sciences allow the application of a personal counselling, in contrary to the general use of standard nutritional diets that was being applied for many years. The personalized nutritional counseling can be used not only to change diet habits and improve life style, but also mainly will permit a better diagnostic of certain diseases, retard the evolution of chronic illnesses, and assist on the treatment of others. Although Nutrigenomics is already available for clinical use, there are still few places applying this tool in the heatlh field [15].

Since the introduction of Nutrigenomics into the “omics” group, there was an upgrade of how physicians and other professionals on evaluating and treating different diseases, especially DCTNs. Nevertheless, there is still a long way to go on Nutrigenomics, as further research needs to be done in order to connect a patient's symptons/diseases with their genetic profile, food diet, and environmental habits.

Moreover, with the knowledge integration of technologies into health sciences, novel business models for personalized nutritional counseling were highlighted, based on the person's DNA. In this way, entities connected to the health system must act in order to regulate these business models in order to preserve the integrity of patients, increasing the performance of the system for nutritional orientation.

Therefore, scientists and professionals of human health sciences can constantly contribute to Nutrigenomics through research and on the development of new tools that can assist on a better quality of life and healthy diet to the population.

## Figures and Tables

**Table 1:** Classification of Ronteltap and colleagues [15], showing the new marketing strategies into nine business models.

Business model	Marketing strategy
Orientation according to the lifestyle of the individual	Shared responsibility between employer and employee, where a healthy lifestyle can contribute to a better productivity.

We are togheter and strong	Improve the lifestyle with the help of social groups.

Gyms	Based on a set of changes into the lifestyle, including physical activities, diet therapies, and use of food supplement, among others.

Do a healthy diet yourself	This model offers a diagnostic tool where, based on the person's food ingestion, it can create a diet plan to improve a healthy diet.

In, out	Similar to the 4th plan reported above. In this, the person describes the food intake but also some phenotype parameters in order to receive diet counseling.

Test and execute until the end	Offers an interactive feedback about a person's health progress, which allows toadjust the diet counseling as needed.

Orientation about the lifestyle	Inclusion of genetic information, data about food intake, and the person's phenotype, in order to obtain a personalized counseling on lifestyle, diet terapy, control of stress activity, and time management.

Face to face	In person treatment, where anthropometric data and food consumption of the cliente are obtained by a face-to-face interview.

We tell you	Use of mass media as a healthy diet education method.
